# Doctors and nurses subjective predictions of 6-month outcome compared to actual 6-month outcome for adult patients with spontaneous intracerebral haemorrhage (ICH) in neurocritical care: An observational study

**DOI:** 10.1016/j.ensci.2023.100491

**Published:** 2023-12-22

**Authors:** Siobhan Mc Lernon, Daniel Frings, Louise Terry, Rob Simister, Simone Browning, Helen Burgess, Josenile Chua, Ugan Reddy, David J. Werring

**Affiliations:** a Stroke Research Centre, University College London, Institute of Neurology, London, UK; b London South Bank University, School of Health and Social Care, London, UK; c National Hospital for Neurology and Neurosurgery, University College London Hospitals NHS Foundation, Queen Square, London, UK; d London South Bank University, School of Applied Sciences, London, UK; e University College London Hospital NHS Foundation Trust, Hyper Acute Stroke Unit, National Hospital for Neurology and Neurosurgery, UK

**Keywords:** Spontaneous intracerebral haemorrhage, Neurocritical care predictions, Prognostication, Modified Rankin Scale (mRS), Outcome

## Abstract

**Background:**

Acute spontaneous intracerebral haemorrhage is a devastating form of stroke. Prognostication after ICH may be influenced by clinicians' subjective opinions.

**Purpose:**

To evaluate subjective predictions of 6-month outcome by clinicians' for ICH patients in a neurocritical care using the modified Rankin Scale (mRS) and compare these to actual 6-month outcome.

**Method:**

We included clinicians' predictions of 6-month outcome in the first 48 h for 52 adults with ICH and compared to actual 6-month outcome using descriptive statistics and multilevel binomial logistic regression.

**Results:**

35/52 patients (66%) had a poor 6-month outcome (mRS 4–6); 19/52 (36%) had died. 324 predictions were included. For good (mRS 0–3) versus poor (mRS 4–6), outcome, accuracy of predictions was 68% and exact agreement 29%. mRS 6 and mRS 4 received the most correct predictions. Comparing job roles, predictions of death were underestimated, by doctors (12%) and nurses (13%) compared with actual mortality (36%). Predictions of vital status showed no significant difference between doctors and nurses: OR = 1.24 {CI; 0.50–3.05}; (*p* = 0.64) or good versus poor outcome: OR = 1.65 {CI; 0.98–2.79}; (*p* = 0.06). When predicted and actual 6-month outcome were compared, job role did not significantly relate to correct predictions of good versus poor outcome: OR = 1.13 {CI;0.67–1.90}; (*p* = 0.65) or for vital status: OR = 1.11 {CI; 0.47–2.61}; *p* = 0.81).

**Conclusions:**

Early prognostication is challenging. Doctors and nurses were most likely to correctly predict poor outcome but tended to err on the side of optimism for mortality, suggesting an absence of clinical nihilism in relation to ICH.

## Highlights

• Doctors and nurses are realistic about the likelihood of a poor outcome but tend to err on the side of optimism for prognosticating ICH mortality suggesting an absence of clinical nihilism.
• Realistic optimism may underpin early prognostication in neurocritical care.
• No sub-group were more accurate than any other when predicting outcome.
• Prognostication is subjective and variable in the first 48 h of ICH admission to neurocritical care highlighting a need for a standardised approach.

## 1 Introduction

Globally, stroke remains the second leading cause of death and the third leading cause of death and disability [1] with a likely increasing disease burden particularly in low-middle income countries [1]. Spontaneous (non-traumatic) intracerebral haemorrhage (ICH) is the most devastating form of stroke and despite being less frequent than ischemic stroke (constituting 27.9% of all new strokes in 2019), it is associated with mortality rates as high as 40% to 50% within 30 days and only 12% to 39% of survivors achieving long-term functional independence [2,3]. Despite the health burden, there are limited definitive treatment options that have improved the mortality and morbidity associated with this stroke type over recent decades when compared to the successes in changing outcomes of ischemic stroke.

Due to the poor outcome after ICH, prognostication is of great importance to guide clinical decision making and risk stratification. However, prognostication after ICH is complex and may be inherently influenced by physicians' subjective impressions and biases which may, in turn, influence future decision-making regarding level of care. Specifically, inaccuracies in prognostication using current prediction models [4,5] might result in premature withdrawal of care [6], thereby creating a self-fulfilling prophecy [7] where the predicted outcome is almost invariably poor [[7], [8], [9]]. Early care-limiting decisions such as DNACPR orders were found to be frequently used in patients with ICH in the acute phase [10] perhaps arising from the presumption of a poor outcome and a lack of proven effective interventions. This suggests that clinical nihilism [11] which has historically pervaded the management of ICH [11,12] may still exist regarding ICH in the UK. However, clinical nihilism might lead to poor outcomes [13] even after adjustment for stroke severity, so avoidance of early withholding or withdrawal of treatment for at least the first two full days of hospitalisation is recommended [14].

There is evidence that bedside clinicians' clinical judgement may be superior to prediction model estimates of ICH prognosis [15,16], although other data suggest variation with potential for both optimism and pessimism [17]. Some studies suggest that doctors might be pessimistic regarding their prognosis, especially for those training in acute care specialities [18]. Variability in clinician prognosis has implications for ICH patient care including variability in treatment and thus outcome. To date, there are limited data on early subjective predictions and how these compare to actual 6-month outcome after treatment of ICH in neurocritical care. To address this, we aimed to [1] evaluate clinicians' early subjective predictions of 6-month outcome for patients with ICH treated in neurocritical care and [2] compare these to actual 6-month outcome.

## 2 Materials and methods

### 2.1 Patient and clinician selection

This study is a single-centre prospective observational cohort study conducted within a neurocritical care department in a neuroscience tertiary referral centre in a multi-site acute, teaching hospital within a large metropolitan area in the southeast of England between September 2018–March 2020. The setting is a high-volume centre for stroke care receiving approximately 100–110 ICH patients (average 9 per month) per year. This ensured that the study setting was representative of the ICH population of patients. Institutional ethics and research board approval was obtained from the UK Heath Research Authority (HRA) in May 2018 (19/HRA/0089) and ratified by London South Bank University Research Ethics Committee in September 2018(HSCSEP/18/05).

Clinicians included doctors and nurses (excluding locums or agency staff) with at least six months' experience of working within a neurocritical care setting who agreed to participate. We invited participants to predict outcomes for all eligible adult patients admitted to neurocritical care with a confirmed spontaneous (non-traumatic) ICH documented by computed tomography (CT) or MRI scan.

Designated audit nurses invited clinicians to subjectively predict 6-month outcome assessed using the modified Rankin Score (mRS) [19] for patients with acute ICH within the first 48 h of admission to neurocritical care by completion of a structured proforma (see supplementary digital). Demographic and clinical characteristics of clinicians and patients were collected. The designated audit nurses ensured that the proformas were completed anonymously, independent of each other by individual clinicians away from the patient's bedside. The early 48-h period for predicting ICH patient outcome was adopted, since this would be before most decisions regarding ongoing level of care for severely-affected patients were made [15]. This time period is a time where physiological stabilisation is optimised for patients with perceived devastating brain injury due to prognostic uncertainty in the acute phase [8,20]. However, to account for potential interdependence despite these controls, we also used a multi-level regression approach which does not assume ratings are independent of one another, rather, it accounts for and controls for shared variance at the patient level.

ICH patient 6-month follow up following hospital discharge was measured via telephone interview as part of the National Institute of Health Research (NIHR) Biomedical Research Centre (BRC)-funded Stroke Investigation in North and Central London (SIGNAL) project. The SIGNAL registry was approved by the UCL Hospitals NHS Foundation Trust Governance Review Board as a Service Evaluation (code: 5–201,920-SE). Since data were collected as part of routine clinical care, the requirement for informed patient consent was waived.

### 2.2 Power calculation

In prior research, five doctors were observed to have a high predictive accuracy (80%) in determining 6-month outcome in mechanically ventilated neurologic patients of which stroke patients were the largest group [21]. Accuracy for predicting a good outcome (mRS 0–3) was 63% and poor outcome (mRS 4–6) 94% [21]. Accuracy for exact agreement between doctors' mRS predictions and actual 6-month mRS was 43% [21]. Based on these findings we conducted a-prori power calculations to determine likely achieved power for an anticipated sample of *n* = 315 observations. Power levels at three possible levels of difference between doctors and nurses were modelled: Assuming that doctors are correct 80% of the time, and nurses correct 60% of the time, a chi-square with a number of 315 would achieve a power of 0.97, with an alpha of 0.05. Assuming that doctors are correct 50% of the time, and nurses correct 30% of the time, a chi-square with a number of 315 would achieve a power of 0.95, with an alpha of 0.05. Assuming that doctors are correct 75% of the time, and nurses correct 60% of the time, a chi-square with an n of 315 would achieve a power of 0.81, with an alpha of 0.05.

## 3 Statistical analysis

The analysis plan was pre-registered in the Open Science Framework (OSF) in January 2020. Ordinal measures were taken initially (mRS 0–6), and then dichotomised for analysis as good (mRS 0–3) or poor (mRS 4–6) outcome as the primary outcome measure. A secondary outcome measure for predictions and actual 6-month outcomes was vital status (alive versus dead). Functional outcome was analysed in this study using dichotomisation of the mRS, which is still the prevailing choice of analysis of an ordinal scale in stroke trials [22]. The literature mostly considers a good outcome as mRS 0–3 [23]; therefore, it was decided to use a mRS dichotomy of 0–3 (good/favourable outcome) as this describes retained independence and ability to walk without assistance, which is likely to be an important and relevant outcome for patients and medical professionals.

Descriptive statistics were used to describe baseline characteristics. A multilevel binomial logistic regression (LR) model employing generalised mixed models in SPSS (version 26) was used to analyse the data. A multilevel analysis was required as the data were interdependent and were clustered between groups (i.e., more than one prediction was made by differing job roles for each individual ICH patient). The multilevel regression model included the patient as a random effect and job role as a fixed effect and accounts for and controls for shared variance at the patient level.

Model statistics were reported along with coefficient estimates and associated 95% confidence levels (CIs) as appropriate.

## 4 Results

### 4.1 Descriptives

A total of 52 patients with ICH were included in the final analysis with 324 early predictions of 6-month outcome. 58% had supratentorial ICH. 57% were male and 43% female. The average age was 60.39 (SD12.19) years. 62% were ≥ 65 years of age. Each eligible ICH patient had between 1 and 11 predictions (Mean 6).

194 (59%) of the predictions were made by doctors with various roles. Critical care fellows (23%) and consultant neuroanaesthetists/neurointensivistists (22%) provided the highest number of predictions. 135 (41%) were from nurses with various job roles. 212 (64.4%) of the predictions were by nurses and doctors who had >10 years' experience (Median 2.00; IQR 2). Table 1 describes 6-month outcome. 1 patient (2%) was lost to follow-up at 6-months. The majority of the cohort had a poor outcome (mRS 4–6) (mRS 0–3){35 (67%) versus 17 (33%)} respectively. Mortality at 6-months was 19 (36%).

**Table 1:** 6-month outcome (mRS 0–6) for 52 patients with ICH.

**modified Rankin Scale (mRS)**	**Patients (n) (%)**
0-no symptoms	3(6%)
1-no significant disability	1(2%)
2-slight disability	5(9%)
3-moderate disability	8(15%)
4-moderately severe disability	9(17%)
5-severe disability	7(13%)
6-dead	19(36%)
Not Known	1(2%)

For the primary outcome of good (mRS 0–3) versus poor (mRS 4–6), 220/324 (68%) predictions were correct. Individual mRS scores, were correctly predicted 93/324 (29%) times for 32 patients which represented 46% of the total number of predictions (203/324). Table 2 describes the number (n) and percentage (%) of the 93 correct predictions. mRS 6 and mRS 4 received the highest number of individual correct predictions (32 and 31, respectively).

**Table 2:** Correct predictions for 32 patients.

**mRS**	**Actual 6-month mRS**	**Total predictions (n)**	**Correct predictions** **(n) (%)**
mRS 0	1	11	2 (2%)
mRS 1	0	0	0 (0%)
mRS 2	3	20	9 (10%)
mRS 3	5	33	13(14%)
mRS 4	9	61	31(33%)
mRS 5	4	25	6 (6%)
mRS 6	10	53	32(34%)
**TOTAL**	**32**	**203**	**93**

**Fig. 1:**
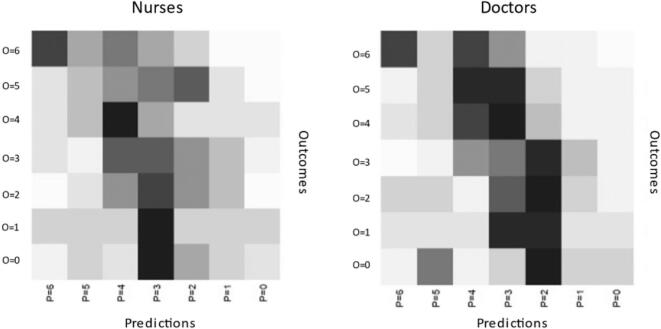
Heat map showing levels of convergence between predictions and outcomes for doctors and nurses. Darker spots indicate more frequent occurrenes of a specific combination. P = predicted mRS at baseline, O = observed actual outcome mRS at 6 months.

Fig. 1 describes the levels of convergence between predictions and 6-month actual outcome, indicating that doctors tend to predict outcome correctly more often than nurses with greater convergence for poor outcome (mRS 4–6). For our secondary outcome (vital status; alive versus dead), 20 patients had incorrect predictions; 14 patients had 72 incorrect predictions for alive and 6 patients had 8 incorrect predictions for death at 6-months.

**Fig. 2:**
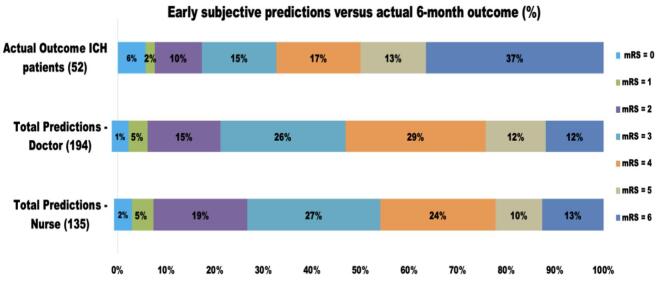
Perceived 6-month functional outcome versus actual 6-month functional outcome. Abbreviations: modified Rankin Scale (mRS).

Fig. 2 shows percentage (%) doctors' (194) and nurses' (135) early subjective predictions of 6-month functional outcome versus actual 6-month functional outcome. Death was underestimated at 6-months, 23 (12%) predictions by doctors and 17(13%) predictions by nurses compared with actual mortality 19 (36%).

Multilevel binomial logistic regression model

### 4.2 Early subjective predictions in first 48 h

The multilevel binomial logistic regression model examined predictions of good outcome (mRS 0–3) versus poor outcome (mRS 4–6) by doctors and nurses with the ICH patient included as a random effect, and job role as a fixed effect. Bootstrapped samples were taken. 95% confidence intervals are reported. The overall model predicted 79.9% of cases. Job role approached but did not reach significance in relation to predictions of good versus poor outcome, *F*(1,322) = 3.56, *p* = 0.06; coefficient = 0.50, *t* = 1.89, OR = 1.65 (CIs; 0.98–2.79; *p* = 0.06). As expected, the patient accounted for a significant amount of the variance, *Z* = 3.046, *p* ≤0.001 in predictions. The same analysis was repeated for predictions of vital status at 6-months. The model predicted 94.1% of cases. Job role did not reach significance in relation to predictions of vital status *F*(1,322) = 0.28; *p* = 0.64) coefficient = 0.21, *t* = 0.47, OR = 1.24 (CIs; 0.50–3.05; p = 0.64).

### 4.3 Comparison of actual 6-month outcome to early predictions

The overall model predicted 77.2% of cases and the number of correct predictions of good and poor 6-month outcome. Job role did not reach significance in relation to the frequency of correct predictions of good versus poor outcome at 6-months, *F*(1,322) =0.21; *p* = 0.65); coefficient = 0.12, *t* = 0.46, OR = 1.13 (CI;0.67–1.90; *p* = 0.65). The model was repeated for frequency of correct predictions for vital status at 6-months. The overall model predicted 94.8% of the cases. Job role did not significantly relate to the frequency of correct predictions of vital status at 6-months. *F*(1,322) = 0.06, *p* = 0.81); coefficient = 0.11, *t* = 0.25, OR = 1.11 (CI; 0.47–2.61; *p* = 0.81).

## 5 Discussion

We found that the majority of patients with ICH had a poor outcome at 6-months following hospital discharge. This finding concurs with existing evidence [[24], [25], [26]]. Our descriptive data showed accuracy of predictions improved when 6-month outcome was dichotomised in contrast to accuracy for exact agreement of ordinal mRS predictions and actual 6-month outcome (68% versus 29% respectively). Earlier studies have also shown low exact (nominal) agreement, 43% [20] and 44% [27] between mRS predictions and actual 6-month mRS. In this study, clinician's exact estimation of 6-month outcome after ICH was lower when using the mRS as an ordinal scale. Correct estimation of 6-month outcome was more frequent when it was dichotomised. The lower number of correct predictions of 6-month functional outcome in each category of the mRS perhaps highlights the difficulty in making a precise 6-month functional outcome prediction. It also highlights the subjectivity of the mRS and its limitations [28]. Although, using ordinal approaches analysing the mRS is favoured, the focus in this study was on how doctors and nurses perceived 6-month outcome after critical care, therefore dichotomous analyses provided results that were easily explained [28].

Our data also indicated that there was variability amongst predictions of outcome by doctors and nurses and incorrect predictions for vital status at 6-months.

These findings highlight the subjective nature of prognosis and the uncertainty that is known to exist following ICH [17,29,30]. This subjectivity aligns with other fields; for instance, studies of prognostic accuracy in palliative care also suggest that clinicians' predictions are frequently inaccurate [31], and variability in prognostication in severe traumatic brain injury also exists [32]. Predicting long-term outcome is therefore challenging as it is nuanced and multifaceted [33] as premorbid status, the timing, intensity, duration and quality of rehabilitation [34], individual preferences, beliefs, values, resilience, and socioeconomic support [35] are all part of the recovery process that determine long-term outcome measures.

Our study showed that job role did not reach significance for correct predictions of good versus poor outcome or for correct predictions of vital status when compared to actual 6-month outcome. This coincides with existing data that have found no sub-group of clinicians to be more accurate than any other [15,31]. However, our correlation “heat map” (Fig. 1) suggests closer agreement for doctors than nurses with greater convergence for predictions of poor outcome (mRS 4–6) and actual 6-month outcome. Combining professional judgement with predictions from prognostic models, could help experienced doctors to provide a more accurate prognosis than nurses [36] as the majority of doctors in our study were experienced in caring for patients with ICH. Clinicians often use their own experience, based on previous patients' outcomes to predict outcome [11]. However, clinical experience-based prognostication may be prone to heuristics and biases [37]. Recognising patterns of disease, experience, intuitive judgement and ‘rule of thumb’ applications are all part of heuristic decision making [37,38]. Doctors with less experience and working in acute settings tend to be more pessimistic [18] and therefore may be more prone to clinical nihilism. A standardised approach to prognostication is needed to guide those with less experience and to limit bias [36]. Exposure to long-term patient outcomes and a multidisciplinary environment are recommended as debiasing strategies [18].

Our results suggest that doctors and nurses caring for patients with ICH were realistic regarding poor 6-month outcome as mRS 4 and mRS 6 were predicted correctly the most often. It is arguable that our study participants shared similar, and realistic, expectations of 6-month outcome for patients with ICH as those that require neurocritical care are a priori the most severely-affected and are unlikely to survive without some level of disability after discharge [25,26]. This was likely to be reflected in their predictions. Importantly, both doctors and nurses, tended to err on the side of optimism for prognosticating ICH mortality which does not support the idea of clinical nihilism in relation to ICH within a neurocritical care setting. This is reassuring, as ICH survivors have a slower rate of recovery than other stroke subtypes [34]. Some patients have initial poor functional outcome but can continue to functionally improve up to 1 year after making it difficult to predict how someone may recover and adapt [39]. Thus, avoiding early pessimistic prognostication and a less nihilistic approach to ICH care is important in the early acute phase. Delaying prognostication until after several days of treatment may improve ability to predict future recovery [33,39].

Early optimism for survival has been reported previously [15,31] and refers to hopefulness and/or a belief that something positive will happen [40,41]. While optimism can be beneficial, clinicians must guard against over-optimism for survival as this may produce unwanted long-term personal, social, cognitive and/or economic consequences. Over-treatment can cause excessive suffering, burden and cost, yet premature withdrawal of treatment can result in patients dying who might otherwise have had acceptable outcomes with appropriate treatment [17].

We hypothesise that the combination of realistic expectations of poor outcome for selected patients and the optimism for survival is perhaps a construct of realistic optimism [40]. Realistic optimism is described as an ability to anticipate good things to happen in the future while taking into account circumstantial factors that may affect the likelihood of outcomes occurring [40,41]. In other words, an acceptance that few severely affected patients with ICH that require neurocritical care are likely to survive without any neurologic deficits [21]. Realistic optimism is associated with greater psychological and physical wellbeing [42] and we suggest that this may be a necessary coping strategy required to work in a highly complex and stressful environment such as neurocritical care with high patient mortality and morbidity, and daily ethical dilemmas [43]. The balance between pessimism and over-optimism i.e., realistic optimism is perhaps a preferable and necessary component of acute stroke care and developing this as a clinical skill may be important in neurocritical care.

Prognostication is important to triage, determine clinical management and to predict the patient's outlook [36]. Surrogates rely on clinicians to provide them with a prognosis with which to make decisions on treatments and goals of care on behalf of the patient as they are often unable to make their own decisions because of the acuity of ICH [17,36]. Therefore, how a prognostic estimate is derived, and then communicated is important as errors in prognostication can have considerable consequences in life and death decisions. Previous studies have included clinical variables such as high illness severity scores, absence of brainstem reflexes, GCS sum score lower than 8, to predict outcome upon critical care admission after stroke [24]. Although clinical variables are useful in prognostic studies, a combination of detailed clinical, laboratory and radiological information as well as clinicians' prognostic estimates may improve predictive accuracy.

Discordant prognostic information can result in surrogate decision-maker distress [17,44]. Clinicians need to acknowledge their uncertainty when giving prognostic estimates so that it can be considered appropriately in shared decision-making [36]. A key component of shared decision making incorporates explaining prognosis in an unbiased way and to acknowledge uncertainty [36]. Providing patients and their families with an early, personalised and realistic assessment of the likelihood of survival and functional outcome after ICH is key to shared decision-making and planning patient care for severely affected ICH patients.

## 6 Study limitations

Our study has several limitations. It involved a single, high capacity, neurocritical care centre, where the majority of participants had >5 years' experience of caring for patients with ICH. This may not be generalisable to other neurocritical care settings. Furthermore, as the neurocritical care model has a unique structure, results may not be generalisable to non-speciality critical care. Future research should include both neurocritical and non-specialist critical care to explore a wider range of clinicians' predictions and to make comparisons between centres.

Our results showed that each individual patient that presented with ICH had a consistent significant effect when doctors and nurses made their early predictions of outcome. This is not surprising and suggests that each patient's individual demographic, clinical and radiological characteristics as well as psychosocial factors were likely to be considered when making early subjective predictions regarding 6-month outcome. However, specific clinical, radiological and patient demographics associated with death after ICH such as decreasing GCS score (≤ 8) and/or a high National Institutes of Health Stroke Scale (NIHSS) score, high illness severity scores, ICH volume and presence and amount of intraventricular haemorrhage [24,25] were not included in this study. Furthermore, subjective factors that doctors' and nurses incorporate into their early subjective predictions of ICH outcome, such as psychosocial aspects of care, religious, cultural or ethnic background, as well as clinicians' personality and psychology [18] may also influence prognosis accuracy and warrants further research.

## 7 Conclusion

This study offers a unique and original insight into a group of clinicians' early subjective predictions and how they compare to actual 6-month outcome. The variability and subjectivity highlighted the challenges of early prognostication after ICH. Doctors and nurses were realistic about the likelihood of a poor outcome after ICH but were optimistic regarding survival at 6-months suggesting an absence of clinical nihilism. The construct of realistic optimism may underpin early prognostication. Further evaluation should try to identify the interplay of patient factors and decisional factors that nurses and doctors consider when making predictions of outcome. This may help understand what makes some clinicians better prognosticators than others so that evidence-based training can be developed. Accurate prognostication is important so that shared decision making about treatment and goals of care on behalf of patients can be optimised.

## Ethical approval

Ethical approval was granted by the Heath Research Authority (HRA) in May 2018 and ratified by London South Bank University Research Ethics Committee in September 2018. Since data were collected as part of routine clinical care, the requirement for informed patient consent was waived.

## Consent of publication

All authors have read and approved the submitted manuscript; the manuscript has not been submitted elsewhere nor published elsewhere in whole or in part. Authorship requirements have been met.

## Author contribution

Dr. Siobhan Mc Lernon (SML) conceived of the presented idea. Professor Frings verified the analytical methods. Professor Werring and Dr. Terry supervised the findings of this work. Simone Browning, Helen Burgess, Josenile Chua collected data. All authors discussed the results and contributed to the final manuscript.

## Availability of data and materials

The datasets used and/or analysed during the current study are available from the corresponding author on reasonable request.

## Funding

This research did not receive any specific grant from funding agencies in the public, commercial, or not-for-profit sectors.

## Address for Reprints

Dr. Siobhan Mc Lernon, 62 Montpelier Rd, Purley, Surrey CR8 2QA, UK. can we use my work address not home address.

## CRediT authorship contribution statement

**Siobhan Mc Lernon:** Writing – review & editing, Writing – original draft, Conceptualization. **Daniel Frings:** Writing – review & editing, Formal analysis. **Louise Terry:** Writing – review & editing, Supervision. **Rob Simister:** Writing – review & editing. **Simone Browning:** Writing – review & editing. **Helen Burgess:** Data curation. **Josenile Chua:** Data curation. **Ugan Reddy:** Writing – review & editing. **David J. Werring:** Writing – review & editing, Supervision.

## Declaration of Competing Interest

None.

## Appendix A Supplementary data

Supplementary material Image 1
