# Supplementary material for: Doctors and nurses subjective predictions of 6-month outcome compared to actual 6-month outcome for adult patients with spontaneous intracerebral haemorrhage (ICH) in neurocritical care: An observational study
Source: eNeurologicalSci. 2023 Dec 22;34:100491. doi: 10.1016/j.ensci.2023.100491 (PMC10809071; doi:10.1016/j.ensci.2023.100491)
Supplement: Supplementary file 1 — Supplementary material [file mmc1.docx]

**Supplementary digital**

###### Proforma for collecting early predictions of 6-month functional outcome

*Proforma for early prediction of spontaneous Intracerebral Haemorrhage (sICH) patient outcome (Note: To be completed within the first* ***48 hours*** *of an eligible ICH patient admission to neurocritical care as part of a service evaluation)*

**Date** and time patient admitted to neurocritical care-----------------------------------------------------

Date and time proforma completed------------------------------------------------------------------------

Please **tick** which one of the following applies to you:

| Nurse |  | Stroke Registrar |  |
| --- | --- | --- | --- |
| Stroke Consultant |  | Critical Care Fellow |  |
| Consultant Neuroanaesthetist |  | Neurosurgeon |  |
| Other (please state) | |  |  |

Clinical Grade -------------------- Clinical Banding -------------------------------

Designation--------------------------------------

Years of experience, **please tick**

| > 6months -1year | 1-2 years | 2-5 years | > 10 years |
| --- | --- | --- | --- |

| Patient name: | Place patient sticker here |
| --- | --- |
| Patient Hospital Number: |  |
| Date of Birth: Admitted from: |  |

Patient diagnosis on admission: ---------------------------------------- Utilising the modified rankin scale (mRS), Please choose what you consider to be the likely outcome for this patient **at six months after hospital discharge Please tick one of the following**

| **0** - No symptoms. |  |
| --- | --- |
| **1** - No significant disability. Able to carry out all usual activities, despite some symptoms |  |
| **2** - Slight disability. Able to look after own affairs without assistance, but unable to carry  out all previous activities |  |
| **3 -** Moderate disability. Requires some help, but able to walk unassisted. |  |
| **4 -** Moderately severe disability. Unable to attend to own bodily needs without assistance,  and unable to walk unassisted |  |
| **5 -** Severe disability. Requires constant nursing care and attention, bedridden, incontinent. |  |
| **6 -** Dead |  |
